# Draft genome sequences of three poultry *Salmonella* Shamba isolates from South Africa

**DOI:** 10.1128/mra.00300-24

**Published:** 2024-07-31

**Authors:** Musafiri Karama, Opeyemi U. Lawal, Valeria R. Parreira, Mitra Soni, Yanhong Chen, Beniamino T. Cenci-Goga, Luca Grispoldi, Janita Greyling, Lawrence Goodridge

**Affiliations:** 1 Veterinary Public Health Section, Department of Paraclinical Sciences, Faculty of Veterinary Science, University of Pretoria, Onderstepoort, South Africa; 2 Canadian Research Institute for Food Safety (CRIFS), Department of Food Science, University of Guelph, Guelph, Ontario, Canada; 3 Department of Veterinary Medicine, Laboratorio di Ispezione Degli Alimenti di Origine Animale, University of Perugia, Perugia, Italy; 4 Department of Veterinary Tropical Diseases, Faculty of Veterinary Science, University of Pretoria, Onderstepoort, South Africa; University of Maryland School of Medicine, Baltimore, Maryland, USA

**Keywords:** *Salmonella *Shamba, sequence, draft genome, poultry house dust

## Abstract

Nontyphoidal *Salmonella enterica* serovars are foodborne pathogens commonly transmitted through poultry products. Draft genome sequences of three *Salmonella enterica* subsp. *enterica* serovar Shamba isolates which were obtained from poultry house dust in South Africa are reported herein.

## ANNOUNCEMENT

Nontyphoidal Salmonellosis is at the top of the list among bacterial foodborne diseases transmitted through poultry products, worldwide. More than 2,600 *Salmonella* serovars have been identified so far, with more than 500,000 *Salmonella* whole-genome sequences deposited in public databases (https://www.ncbi.nlm.nih.gov/datasets/genome/?taxon=590). However, whole-genome sequences of a number of *Salmonella* serovars such as Shamba remain rare or unavailable. *Salmonella* Shamba has been previously associated with food poisoning and isolated from symptomless animal and human carriers (1, 2). This work presents the draft genome sequences of three *Salmonella* Shamba poultry isolates. The isolates were cultured from poultry house dust samples which were collected in 1999, 2000, and 2001 in South Africa.

*Salmonella* was isolated as described previously (3). Briefly, 25 g of poultry house dust was cultured in 225 mL buffered peptone water for 16–18 hours at 37°C. A 100 µL aliquot of buffered peptone water was inoculated and incubated into 10 mL Rappaport-Vassiliadis medium at 42°C for 24 hours before plating on XLD medium for 24 hours at 37°C. Presumptive *Salmonella* colonies were initially identified by Gram staining, catalase, oxidase, and spot indole tests. The *Salmonella* status of presumptive isolates was verified and confirmed by the API10S and PCR (4). *Salmonella* was serotyped by the Kauffman-White Scheme (5).

Before whole-genome sequencing, frozen *Salmonella* isolates were resuscitated into tryptic soy broth for 24 hours at 35°C, followed by streaking on *Salmonella Shigella* Agar and purification on Brilliance *Salmonella* Agar Base. Purified colonies were identified by VITEK (Biomerieux, Canada). DNA was extracted from pure colonies by the DNeasy Blood and Tissue Kit (Qiagen, Hilden, Germany). Sequencing was carried out as described previously (3, 6). Briefly, DNA libraries were prepared using the Illumina DNA Prep Tagmentation Kit and Integrated DNA Technologies for Illumina DNA/RNA unique dual indexes (3, 6). The Illumina MiniSeq system was used to perform paired-end (2 ×  150 bp) sequencing. Default parameters were used in all bioinformatics tools. Pre-processing of raw reads was performed by FastQC v0.11.9 (https://github.com/s-andrews/FastQC) and Trimmomatic v0.39 (7). SKESA v2.4.0 was used for *de novo* assembly of reads with quality scores above 20 (8). Assembly quality was assessed with QUAST v5.2 (9), and genome annotation was performed using the NCBI Prokaryotic Genome Annotation Pipeline v6.6 (10). The isolates were serotyped and the sequence types were identified by SISTR v1.0 (11) and the PubMLST scheme, respectively (12). Antimicrobial resistance encoding genes and plasmid types were identified using the CARD database (13), and MOB-suite v3.1 (14), respectively.

Genome sequencing revealed that the three *Salmonella* isolates belonged to serotype *Salmonella* Shamba. Genome annotation data regarding genome size, number of reads and contigs, protein-coding genes, tRNAs and ncRNAs, coverage, % GC content, plasmid presence, and antimicrobial resistance gene profiles for the three *Salmonella* isolates are summarized in Table 1. All three *Salmonella* Shamba isolates possessed one CRISPR Array each.

**TABLE 1:** Summary of sequence metrics of three *Salmonella* Shamba isolates recovered from poultry house dust in South Africa

ID	Collection date and origin	Numberofcontigs	Numberofreads	Genome	Proteincodinggenes	%GC	*N*_50_ (bp)	Coverage	Plasmid type	tRNAs	ncRNAs	AMR gene profile	Assembly and SRA accession numbers
*Salmonella* Shamba **SE-110**	1999 Poultry house dust	26	1,626,621	4,596,393	4,203	52.3	395,585	94.08×	None	70	9	*aac(6')-Iaa, mdtK, sdiA, fosA, golS, mdsABC*	JAWDKU000000000.1 SRR26197580
*Salmonella* Shamba **SE-135**	2000 Poultryhouse dust	29	1,568,060	4,598,475	4,202	52.3	477,344	89.91×	None	72	9	*aac(6')-Iaa, mdtK, sdiA, fosA, golS, mdsABC*	JAWDKT000000000.1 SRR26197579
*Salmonella* Shamba **SE-138**	2001 Poultryhouse dust	29	1,640,609	4,703,673	4,314	52.2	329,177	91.03×	lncI-gamma/K	71	9	*aac(6')-Iaa, mdtK, sdiA, fosA, golS, mdsABC, TEM-1, sul2*	JAWDKS000000000.1 SRR26197578

Overall, the draft genomes described in this report will be an indispensable resource for understanding the evolutionary and functional genomics of *Salmonella* Shamba.

## Data Availability

Whole-genome sequences for *Salmonella* Shamba SE-110, SE-135, and SE-138 were deposited in the DDBJ/ENA/GenBank database under the accession numbers JAWDKU000000000.1, JAWDKT000000000.1, JAWDKS000000000.1 and the SRA accession numbers SRR26197580, SRR26197579 and SRR26197578.
